# Interaction of neuropilin-1 and hepatocyte growth factor/C-Met pathway in liver fibrosis progression in hepatocyte-specific NRP-1 knockout mice

**DOI:** 10.1007/s00535-025-02262-8

**Published:** 2025-05-26

**Authors:** Han Ding, Huanran Lv, Minghao Sui, Xinyu Wang, Yanning Sun, Miaomiao Tian, Shujun Ma, Yuchan Xue, Miao Zhang, Xin Wang, Jianni Qi, Le Wang, Qiang Zhu

**Affiliations:** 1 Department of Gastroenterology, Shandong Provincial Hospital Affiliated to Shandong First Medical University, No. 324, Jingwu Weiqi Road, Huaiyin District, Jinan City, Shandong Province China; 2 Department of Gastroenterology, Shandong Provincial Hospital, Cheeloo College of Medicine, Shandong University, No. 324, Jingwu Weiqi Road, Huaiyin District, Jinan City, Shandong Province China; 3 Urology Department, Shandong Provincial Hospital, Cheeloo College of Medicine, Shandong University, No. 324, Jingwu Weiqi Road, Huaiyin District, Jinan City, Shandong Province China; 4 Department of Radiology, Shandong Provincial Hospital Affiliated to Shandong First Medical University, No. 324, Jingwu Weiqi Road, Huaiyin District, Jinan City, Shandong Province China; 5 Department of Ultrasound, Shandong Provincial Hospital Affiliated to Shandong First Medical University, No. 324, Jingwu Weiqi Road, Huaiyin District, Jinan City, Shandong Province China; 6 Department of Key Laboratory, Shandong Provincial Hospital Affiliated to Shandong First Medical University, No. 324, Jingwu Weiqi Road, Huaiyin District, Jinan City, Shandong Province China; 7 Department of Geriatrics, Department of Geriatric Gastroenterology, Shandong Provincial Hospital Affiliated to Shandong First Medical University, No. 324, Jingwu Weiqi Road, Huaiyin District, Jinan City, Shandong Province China; 8 Department of Infectious Diseases, Shandong Provincial Hospital Affiliated to Shandong First Medical University, No. 324, Jingwu Weiqi Road, Huaiyin District, Jinan City, Shandong Province China

**Keywords:** c-Met receptor, Hepatocyte growth factor, Liver fibrosis, Neuropilin-1, Transcription factors

## Abstract

**Background:**

Hepatocyte growth factor (HGF)/c-Met signaling critically influences liver fibrosis, but its interaction with neuropilin-1 (NRP-1) in hepatocytes remains unclear. We investigated the role of hepatocyte-specific NRP-1 deletion in liver fibrosis progression and its relationship with the HGF/c-Met pathway.

**Methods:**

Hepatocyte-specific NRP-1 knockout mice were generated using the Cre-lox system, and liver fibrosis was induced by carbon tetrachloride injections or a methionine- and choline-deficient diet. Fibrosis severity, hepatocyte injury, and cytokine secretion were evaluated via histology, biochemical assays, and molecular analyses in isolated hepatocytes. In vitro experiments were conducted in primary hepatocytes and Huh7 cells using lentiviral overexpression and knockdown of NRP-1. Chromatin immunoprecipitation and dual-luciferase reporter assays were performed to analyze transcription factor binding to the NRP-1 promoter.

**Results:**

Hepatocyte NRP-1 expression increased significantly during liver fibrosis and was positively correlated with HGF/c-Met expression and fibrosis severity. In vivo, NRP-1 inhibition reduced extracellular matrix accumulation and abnormal angiogenesis in Alb-Cre NRP-1^f/f^ mice. In vitro, NRP-1 blockade inhibited c-Met activation and reduced transforming growth factor-beta and vascular endothelial growth factor secretion in hepatocytes. NRP-1 functioned as a co-receptor for HGF/c-Met, with HGF upregulating NRP-1 expression at transcript and protein levels. NRP-1 promoted fibrosis through the Met/extracellular signal-regulated kinase pathway. Furthermore, HGF increased retinoic acid receptor alpha expression, promoting NRP-1 transcription.

**Conclusions:**

HGF-induced upregulation of hepatocyte NRP-1, mediated by RARA binding to its promoter, drives liver fibrosis through c-Met pathway activation, highlighting NRP-1 as a potential therapeutic target for liver fibrosis.

**Supplementary Information:**

The online version contains supplementary material available at 10.1007/s00535-025-02262-8.

## Introduction

Liver fibrosis is a common feature of chronic liver injury that can progress to end-stage liver disease, including cirrhosis, hepatocellular carcinoma, and liver failure [1]. It is a dynamic process driven by the activation of hepatic stellate cells (HSCs), leading to extracellular matrix accumulation, and by hepatic sinusoidal endothelial cells (HSECs), contributing to pathological angiogenesis [2–4].

Hepatocytes, the predominant parenchymal liver cells, are pivotal in initiating liver fibrosis as a primary source of profibrotic and proangiogenic factors during disease progression [5–8]. Targeting hepatocyte pathways may offer potential therapeutic strategies to inhibit or reverse early-stage liver fibrosis [9].

Neuropilin-1 (NRP-1) is a glycoprotein critical in tumor progression, angiogenesis, metastasis, and cell proliferation [10–12]. NRP-1 functions as a co-receptor on cell surfaces, forming complexes with receptors such as the platelet-derived growth factor receptor (PDGFR) and vascular endothelial growth factor receptor (VEGFR), facilitating NRP-1/PDGFR and NRP-1/VEGFR interactions [13–16]. Our previous studies revealed that NRP-1 contributes to hepatic fibrosis by functioning as a co-receptor for PDGF-B and transforming growth factor-beta (TGF-β) in HSCs [17]. Other studies have shown that NRP-1 enhances angiogenesis in HSECs during liver fibrosis as a co-receptor of VEGFR2 [18] and that hepatocyte NRP-1 promotes the proliferation and migration of hepatocellular carcinoma [19–21]. However, the mechanism through which hepatocyte NRP-1 promotes liver fibrosis remains unclear and requires further investigation.

Hepatocyte growth factor (HGF) regulates hepatocyte function, binding to its receptor c-Met to trigger intracellular signaling that modulates hepatocyte activity [22–24]. HGF is abundantly expressed in hepatocytes and is upregulated following liver injury, promoting hepatocyte proliferation, growth, and survival [25, 26]. The HGF/c-Met pathway also contributes in to chronic liver disease (CLD) [27]. Deregulated c-Met activation can lead to adverse effects, with elevated *MET* phosphorylation correlating with poorer outcomes and indicating the severity of liver disease [28]. The HGF/c-Met axis further contributes to disease onset, proliferation, invasion, and metastasis [29–31], underscoring its significance in hepatic pathophysiology. Additionally, studies have shown that NRP-1 functions as a co-receptor for c-Met in other malignancies, such as gastric cancer and cholangiocarcinoma, where it facilitates tumor progression [13]. However, the role of NRP-1 in hepatocytes and its potential interaction with HGF/c-Met in exacerbating liver fibrosis remains unknown.

Our previous studies showed that NRP-1 expression is significantly upregulated in hepatocytes during fibrosis, correlating with HGF, c-Met expression, and fibrosis severity. NRP-1 promotes fibrosis by increasing TGF-β and VEGF release and amplifying the HGF/c-Met signaling pathway. HGF further upregulates NRP-1 through the transcription factor RARA, exacerbating liver fibrosis. Given its key role in fibrosis pathogenesis, NRP-1 is a promising target for diagnostic and therapeutic interventions. Targeting NRP-1 in hepatocytes could effectively inhibit or reverse liver fibrosis.

We aimed to investigate whether the NRP-1 and HGF/c-Met pathways interact to drive liver fibrosis progression. We hypothesize that HGF upregulates NRP-1 via RARA binding to its promoter, facilitating fibrosis progression through c-Met activation.

## Methods

### Ethical approval

All procedures were approved by the Ethics Committee of Shandong Provincial Hospital, affiliated with Shandong First Medical University in Jinan, China (Nos. 2023–037 and SWYX2024-512). This study was conducted in accordance with the Declaration of Helsinki for human experiments and the ARRIVE guidelines for animal experiments. Informed consent was obtained from all participants before the study began.

### Patients

Five liver fibrosis tissue samples were obtained from surgical waste and diagnosed through imaging, clinical examination, and histological evaluation. Additionally, five normal liver tissue samples collected from regions unaffected by hepatic injury were similarly obtained from surgical waste.

### Animal experiments

We used Cre-lox technology to generate mice with hepatocyte-specific NRP-1 deletion by backcrossing NRP-1 flox/flox mice with Alb-Cre mice. NRP-1^f/f^ (RRID: IMSR_JAX:005247) was obtained from The Jackson Laboratory (Hancock, ME, USA), and Alb-Cre mice were sourced from GemPharmatech (Nanjing, China). The primers used for mouse genotyping are provided in Online Resource 1.

To mimic the clinical presentation of liver fibrosis, two murine fibrosis models were used:

CCl₄-induced liver fibrosis model: Eight-week-old male NRP-1^f/f^ and Alb-Cre NRP-1^f/f^ mice received intraperitoneal injections of CCl₄ (5 mL/kg, CCl₄: oil = 1:3) twice weekly for 4 or 8 weeks. Control animals received olive oil injections. Non-alcoholic steatohepatitis (NASH)-induced liver fibrosis model: Male NRP-1^f/f^ and Alb-Cre NRP-1^f/f^ mice were fed a methionine-choline-deficient (MCD) diet (Xiaoshu Youtai, China) for either 6 or 12 weeks to induce NASH-associated fibrosis. Control mice were fed a methionine-choline-sufficient diet (Xiaoshu Youtai, China), which served as the dietary control.

### Isolation of primary liver cells

Primary hepatocytes, liver sinusoidal endothelial cells (LSECs), HSCs, and Kupffer cells were isolated and cultured as follows:

Hepatocytes: Isolated and cultured as previously described [32].

LSECs: Isolated by Percoll gradient centrifugation (900×*g*, 20 min, 4 °C) and CD146+ magnetic-activated cell sorting (MACS) (Miltenyi Biotec, Germany, #130-092-007), then cultured in endothelial cell growth medium (ECGM) with 10% fetal bovine serum (FBS)(Gibco, USA).

HSCs: Isolated from the upper layer of an 11.5% Nycodenz (Sigma-Aldrich, USA) gradient and cultured in Dulbecco's Modified Eagle Medium with 10% FBS. Activated HSCs were obtained by treating cells with 10 ng/mL TGF-β (PeproTech, USA) for 48 h.

Kupffer Cells: Enriched by F4/80+ MACS (Miltenyi Biotec, Germany, #130-110-443) and cultured in RPMI-1640 medium with 10% FBS.

### Cell culture, reagents, and antibodies

Huh7 cells (Procell, Wuhan, China) were cultured in Dulbecco’s Modified Eagle Medium (GibcoBRL, Grand Island, NY, USA), supplemented with 10% FBS (Gibco® Sera, AUS), 100 mg/mL penicillin G, and 50 μg/mL streptomycin (Biological Industries, Israel). Cells were maintained at 37 °C in a 5% CO₂ incubator. The antibodies and small molecule inhibitors used in this experiment are listed in Online Resource 2.

### Cell transfection

Lentiviruses were used to either overexpress or downregulate NRP-1 and RARA in Huh7 cells. The modified lentiviruses were obtained from GeneChem (Shanghai, China). The transfection efficiency was evaluated using real-time polymerase chain reaction (RT-PCR) and Western blotting (WB).

### Quantitative RT-PCR (qRT-PCR)

Total RNA was isolated from cells or liver tissue using RNAiso Plus. Reverse transcription was performed using an RT reagent kit and genomic DNA Eraser (Takara, Japan). RT-qPCR was conducted using a LightCycler RT-PCR System (Roche Diagnostics, USA) and the TB Green Premix Ex Taq II Kit (Takara). Gene expression was normalized to GAPDH levels. Specificity was confirmed by melting curve analysis. Quantitative analysis used the comparative (ΔΔCT) method, with results expressed as fold changes relative to the control. Primer sequences are in Online Resource 1.

### Co-immunoprecipitation (CoIP) and WB analysis

Co-IP and WB were conducted per a previously described protocol [33]. WB data were analyzed using ImageJ software developed by the National Institutes of Health (Bethesda, MD, USA). Glyceraldehyde-3-phosphate dehydrogenase was used as an internal control on the same membrane.

### Enzyme-linked immunosorbent assay (ELISA) and biochemical assays

The concentrations of human VEGF-A (KE00216, Proteintech) and human TGF-β1 (KE00002, Proteintech) in cell culture supernatants were quantified using commercially available ELISA kits, following the manufacturer’s instructions. Serum concentrations of alanine aminotransferase (ALT) and aspartate aminotransferase (AST) were measured using ALT and AST Assay Kits from NanJing JianCheng Bioengineering Institute (Nanjing, China), following the manufacturer’s instructions.

### Histopathologic evaluation and immunohistochemistry

Hepatic samples obtained from biopsies or surgical procedures were fixed in formalin, embedded in paraffin, and sectioned into 5-μm slices. Immunohistochemical staining was performed as described previously [33].

### Immunofluorescent staining

Liver tissue samples were prepared, and immunofluorescence staining was performed following the standard procedure (Servicebio).

### Promoter reporter and dual-luciferase assays

Promoter assays were conducted to elucidate the mechanism by which RARA regulates the expression of NRP-1. The promoter-driven luciferase reporter plasmid for RARA was ligated with full-length human RARA complementary DNA. Luciferase reporter plasmids containing wild-type or mutant NRP-1 promoter sequences were generated from GeneChem Co. Cells were co-transfected with these reporter plasmids and the pEnter-RARA vector using Lipofectamine 3000 (Invitrogen, CA, USA). Luciferase assays were performed 48 h post-transfection using a luciferase reporter assay kit (Promega, Madison, WI, USA), with firefly activity normalized to Renilla activity.

### ChIP assay and ChIP-qPCR

Primary mouse hepatocytes and Huh7 cells were fixed with 1% formaldehyde in PBS for 10 min at room temperature to crosslink protein-DNA complexes, followed by quenching with 1.25 M glycine. After washing, cells were lysed in lysis buffer containing 1% SDS, 50 mM Tris–HCl (pH 8.1), 5 mM EDTA, and protease inhibitors for 1 h on ice. Chromatin was sheared to approximately 300 bp by sonication and centrifuged at 4 °C, 4,000 rpm for 10 min. The supernatant was diluted with buffer and incubated overnight at 4 °C with anti-IgG or anti-RARA antibodies. Dynabeads™ Protein G were added, and incubation continued for 4 h at 4 °C. After extensive washing, both input and immunoprecipitated chromatin were reverse crosslinked at 65 °C for 12 h. DNA was purified using the Qiagen MinElute PCR Purification Kit, and ChIP-qPCR was performed with the SimpleChIP® Plus Enzymatic Chromatin IP Kit. Primer sequences for ChIP-qPCR are listed in Online Resource 1.

### Statistical analysis

Continuous variable data are reported as mean ± standard deviation and categorical variables as frequency or percentage. Student’s t-test or one-way analysis of variance was used to examine significant differences between groups based on a minimum of three independent experiments. Spearman’s correlation coefficients were calculated to characterize the relationships between parameters. Statistical significance was set at a *p* value of < 0.05.

## Results

### Upregulation of NRP-1 in hepatocytes correlates with HGF, c-Met expression, and fibrosis severity

**Fig. 1:**
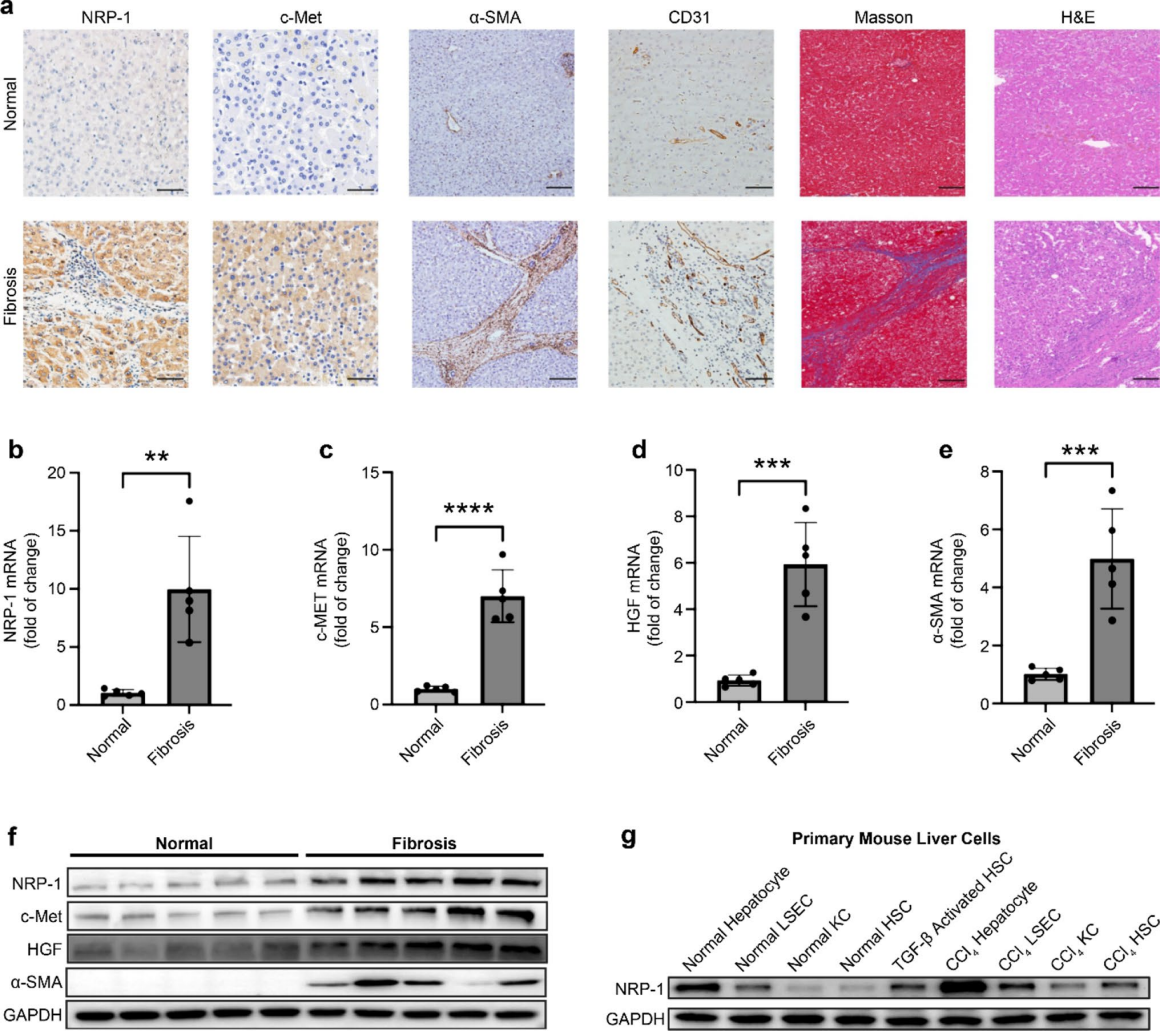
NRP-1 and c-Met expression are elevated in liver fibrosis. (a) Representative immunohistochemistry images of human liver tissues show NRP-1 (×400; scale bar: 50 μm), c-Met (×400; scale bar: 50 μm), α-SMA (×100; scale bar: 200 μm), CD31 (×200; scale bar: 100 μm), hematoxylin and eosin (H&E) staining (×100; scale bar: 200 μm), and Masson’s staining (×100; scale bar: 200 μm) for normal (upper panels, n = 5) and cirrhotic (lower panels, n = 5) groups. NRP-1 and c-Met localized in hepatocytes, CD31 in the microvascular intima, and α-SMA in fibrotic scar tissue. (b–e) qRT-PCR analysis showed significantly higher NRP-1, c-Met, HGF, and α-SMA mRNA levels in cirrhotic liver samples than in normal tissues. (f) Western blotting confirmed increased NRP-1, c-Met, HGF, and α-SMA protein levels in cirrhotic liver samples compared to controls. (g) Western blot analysis of NRP-1 expression was performed in primary hepatocytes, LSECs, Kupffer cells, and HSCs (quiescent and TGF-β-stimulated) from normal and fibrotic mouse livers

NRP-1 expression was significantly higher in human liver fibrosis than in normal tissues (Fig. 1a). Fibrotic livers showed increased expression of NRP-1 and c-Met, consistent with immunohistochemistry results (Fig. 1b, 1c, 1f). HGF levels also increased during fibrosis progression (Fig. 1d, 1f).

To identify the cellular sources of NRP-1, primary hepatocytes, LSECs, Kupffer cells, and HSCs were isolated from both normal and fibrotic mouse livers. WB analysis revealed that hepatocytes exhibited the highest NRP-1 expression under normal conditions. After fibrosis induction, NRP-1 expression increased across all cell types, with hepatocytes remaining the primary source. Additionally, TGF-β-induced HSC activation upregulated NRP-1, although levels remained significantly lower than those in hepatocytes (Fig. 1g). These findings suggest that hepatocytes are the primary contributors to NRP-1 expression in fibrotic livers.

**Fig. 2:**
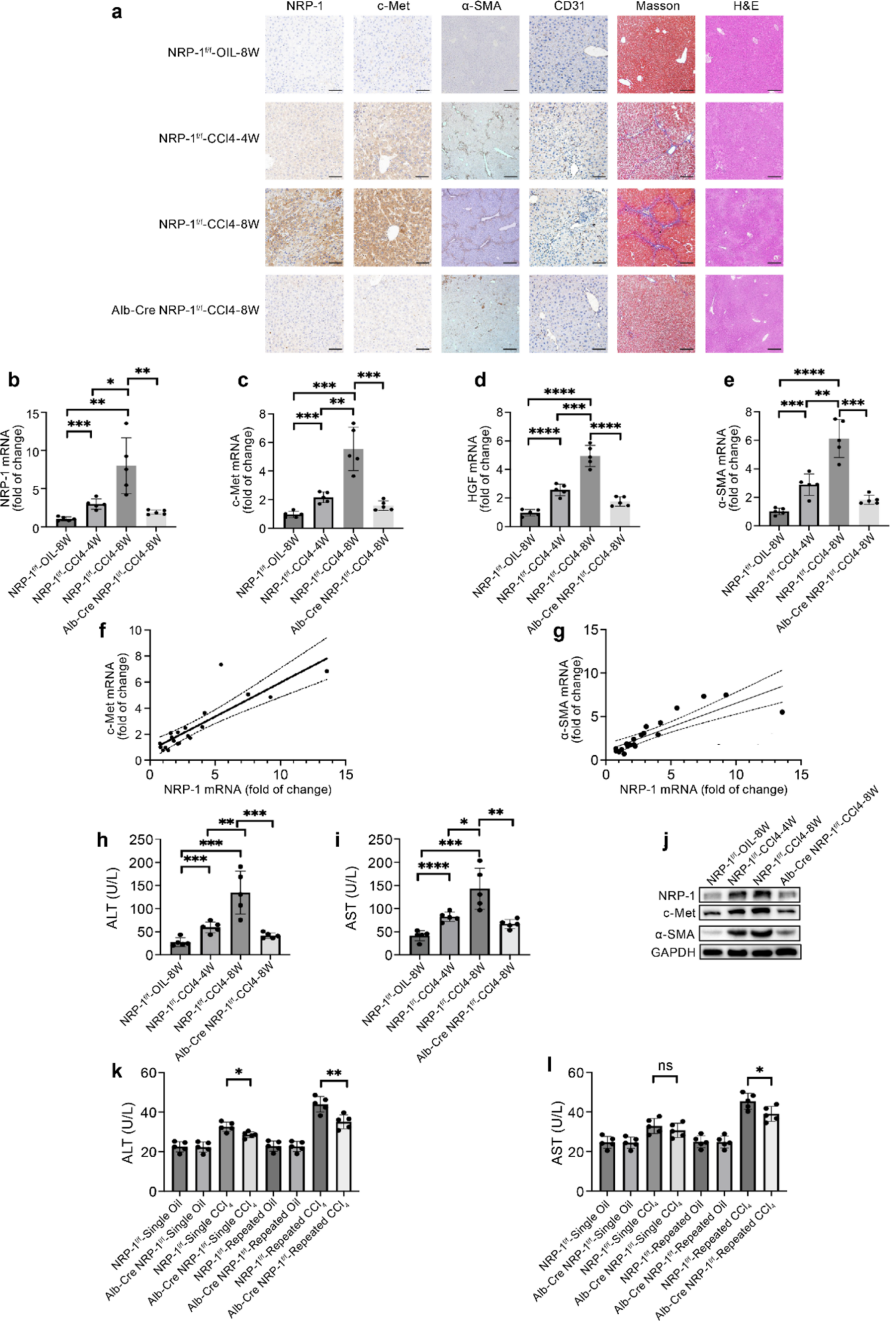
Knocking out NRP-1 ameliorates liver fibrosis in hepatocyte-specific NRP-1-knockout mice induced by CCl₄. (a) Representative histological and immunohistochemical staining of liver tissues from CCl₄-treated mice, showing NRP-1 (×400, scale bar: 50 μm), c-Met (×400, scale bar: 50 μm), α-SMA (×50, scale bar: 400 μm), and CD31 (×400, scale bar: 50 μm). H&E (×100, scale bar: 200 μm) and Masson’s trichrome staining (×100, scale bar: 200 μm) were used to assess fibrosis and collagen deposition. (b–e) qRT-PCR analysis showed significantly higher NRP-1, c-Met, HGF, and α-SMA mRNA levels in fibrotic liver samples. (f, g) NRP-1 mRNA levels correlated significantly with c-Met in human liver samples (*p* < 0.001, *R*^2^ = 0.7421) and with α-SMA in mouse liver samples (*p* < 0.001, *R*^2^ = 0.7087). (h, i) Serum ALT and AST levels increased with fibrosis severity but were significantly reduced after hepatocyte-specific NRP-1 knockout. (j) Western blot analysis of NRP-1, c-Met, and α-SMA expression in normal, CCl₄-induced fibrosis, and NRP-1 knockout CCl₄-fibrosis groups. (k, i) Serum ALT and AST levels are shown in CCl₄-treated and oil-treated mice. *p < 0.05, **p < 0.01, ***p < 0.005, ****p < 0.001. ALT, alanine aminotransferase; AST, aspartate aminotransferase

To assess the role of NRP-1in fibrosis, we established a hepatocyte-specific NRP-1 knockdown mouse model. Immunohistochemical staining confirmed that NRP-1 expression increased with fibrosis severity in the NRP-1^f/f^-CCl4-4W and NRP-1^f/f^-CCl4-8W groups, whereas Alb-Cre NRP-1^f/f^ mice exhibited significantly reduced NRP-1 expression, confirming successful knockout (Fig. 2a). H&E, Masson’s, and immunohistochemical staining revealed that fibrosis and pathological angiogenesis worsened with prolonged CCl₄ exposure but were markedly attenuated in NRP-1 knockdown mice (Fig. 2a). To validate these findings in another fibrosis model, we generated hepatocyte-specific NRP-1 knockout mice under NASH-induced fibrosis (MCD diet, 12 weeks). Histological analysis revealed significantly reduced fibrosis and angiogenesis in Alb-Cre NRP-1^f/f^ mice compared to controls, with less collagen deposition and lower α-SMA and CD31 expression (Supplementary Fig. 1b). These results mirrored those from the CCl₄ model, reinforcing the anti-fibrotic effects of hepatocyte-specific NRP-1 deletion across different etiologies.

Furthermore, α-SMA, NRP-1, c-Met, and HGF mRNA levels increased with fibrosis progression, while NRP-1 knockdown significantly reduced their expression (Fig. 2b–e). NRP-1 expression correlated positively with c-Met and α-SMA during fibrosis (Fig. 2f–g), a trend validated in primary liver cells. Prolonged CCl₄ exposure markedly increased NRP-1, c-Met, and α-SMA levels in liver tissues, while NRP-1 knockout significantly reduced fibrosis and suppressed c-Met expression (Fig. 2j). Similarly, in the NASH model, NRP-1 deletion led to reduced fibrosis and lower c-Met expression (Supplementary Fig. 1e).

### NRP-1 promoted the release of TGF-β and VEGF in hepatocytes

Damaged hepatocytes release cytokines that activate HSCs and HSECs, contributing to liver fibrosis. Serum ALT and AST levels, markers of hepatocyte damage, increased with the severity of fibrosis but were significantly reduced following hepatocyte-specific NRP-1 deletion (Fig. 2h–i). Similar results were observed in the NASH-induced fibrosis model, where NRP-1 knockout also decreased ALT and AST levels (Online Resource 3, Supplementary Fig. 1c–d).

**Fig. 3:**
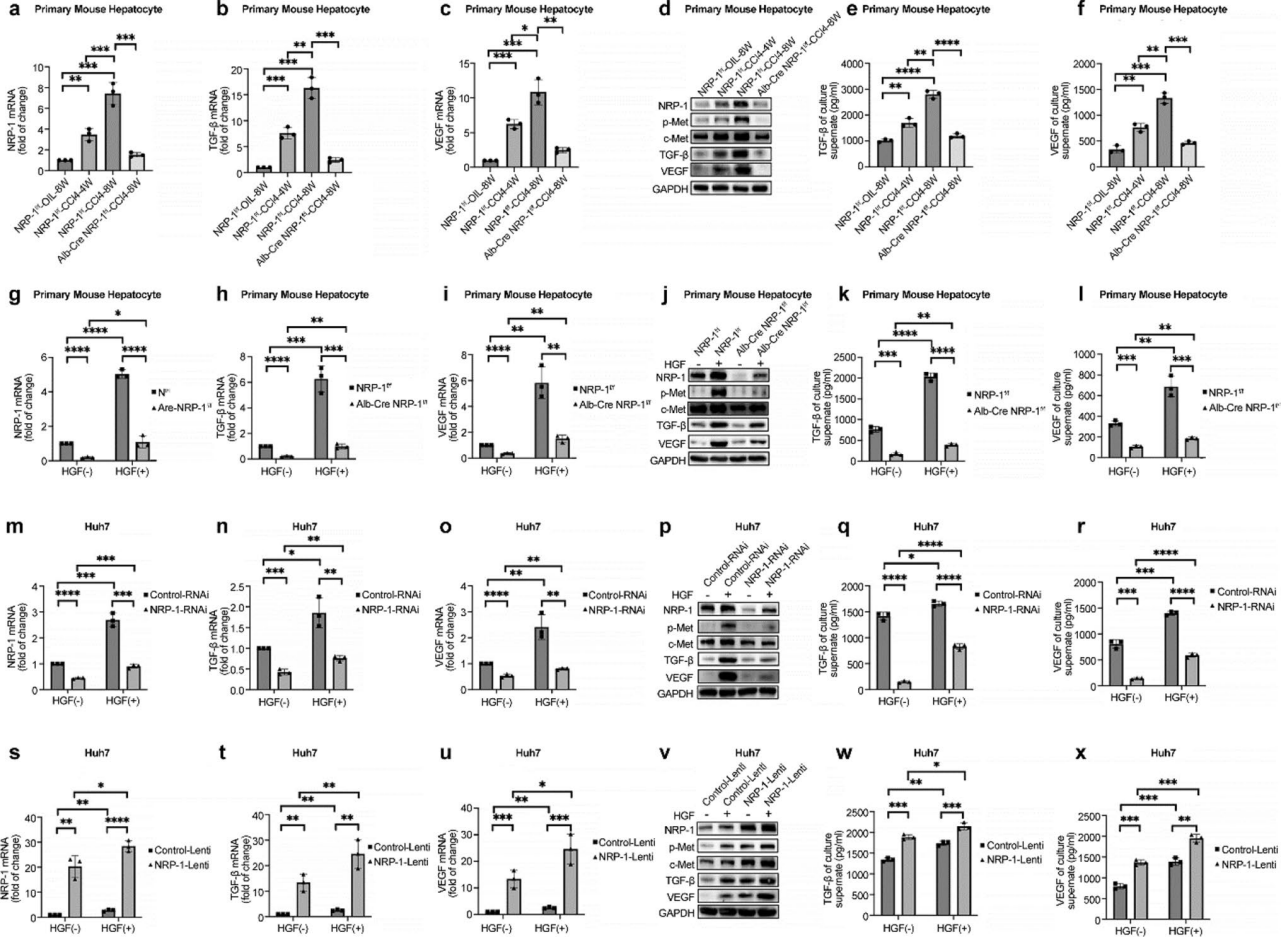
NRP-1 promotes the release of TGF-β and VEGF in hepatocytes. (a–c) qRT-PCR analysis showing significantly elevated mRNA levels of NRP-1, TGF-β, and VEGF in primary hepatocytes from fibrotic mice, with NRP-1 deletion markedly reducing these levels. (d) Western blot analysis demonstrating increased NRP-1, p-Met, TGF-β, and VEGF protein levels in primary hepatocytes from CCl₄-treated mice, correlating with fibrosis severity. Hepatocyte-specific NRP-1 knockout reduced their expression. (e–f) ELISA showing higher TGF-β and VEGF secretion in fibrotic hepatocytes, significantly reduced following NRP-1 deletion. (g–i) qRT-PCR analysis of NRP-1, TGF-β, and VEGF mRNA in primary hepatocytes isolated from NRP-1^f/f^ and Alb-Cre NRP-1^f/f^ mice. (j) Western blot analysis of NRP-1, p-Met, TGF-β, and VEGF protein levels in hepatocytes from NRP-1^f/f^ and Alb-Cre NRP-1^f/f^ mice. (k–l) ELISA confirming changes in TGF-β and VEGF secretion in hepatocytes from NRP-1^f/f^ and Alb-Cre NRP-1^f/f^ mice. (m–o) qRT-PCR analysis of NRP-1, TGF-β, and VEGF mRNA in Huh7 cells transfected with NRP-1-RNAi or Control-RNAi. (p) Western blot analysis of NRP-1, p-Met, TGF-β, and VEGF protein levels in Huh7 cells. (q–r) ELISA showing reduced TGF-β and VEGF secretion in NRP-1-RNAi-transfected Huh7 cells. (s–u) qRT-PCR analysis of NRP-1, TGF-β, and VEGF mRNA in Lenti-NRP-1 and Lenti-Control groups. (v) Western blot analysis of NRP-1, p-Met, TGF-β, and VEGF protein expression in Lenti-NRP-1 and Lenti-Control groups. (w–x) ELISA showing increased TGF-β and VEGF secretion in Lenti-NRP-1-overexpressing cells. *p < 0.05, **p < 0.01, ***p < 0.005, ****p < 0.001

To further investigate this protective effect, ALT and AST levels were measured in Alb-Cre NRP-1^f/f^ and NRP-1^f/f^ mice after a single CCl₄injection. ALT was significantly lower in NRP-1 knockout mice after one injection (p = 0.0116), while AST showed a decreasing trend without statistical significance (*p* = 0.3740). After two injections, ALT and AST levels were significantly reduced (ALT: *p* = 0.0057; AST: *p* = 0.0322), suggesting that NRP-1 deletion confers cumulative protection against hepatocyte damage and fibrosis progression (Fig. 2k–l). Primary hepatocytes were isolated from fibrotic and control mice to confirm NRP-1’s role in hepatocyte-derived TGF-β and VEGF secretion. WB and qRT-PCR showed significantly higher TGF-βand VEGF levels in fibrotic hepatocytes, which were markedly reduced in NRP-1–deleted hepatocytes (Fig. 3b–d). ELISA of hepatocyte supernatants further confirmed the increased secretion of TGF-β and VEGF in fibrotic hepatocytes and the decreased secretion following NRP-1 knockout (Fig. 3e–f). To validate these findings in vitro, NRP-1 gain- and loss-of-function studies were performed in Huh7 cells using lentiviral vectors. Overexpression of NRP-1 significantly increased TGF-β and VEGF levels, while NRP-1 knockdown had the opposite effect (Fig. 3m–r, 3s–x). Notably, modulation of NRP-1 also influenced c-Met protein levels, with knockout suppressing c-Met activation and reducing TGF-β and VEGF secretion in primary hepatocytes (Fig. 3g–l). Conversely, NRP-1 overexpression in Huh7 cells enhanced c-Met activation and further increased TGF-β and VEGF secretion (Fig. 3t–x), indicating NRP-1 regulates c-Met signaling, promoting fibrosis progression through TGF-β and VEGF secretion.

### Hepatocyte NRP-1 worked as a co-receptor of c-Met in liver fibrosis

**Fig. 4:**
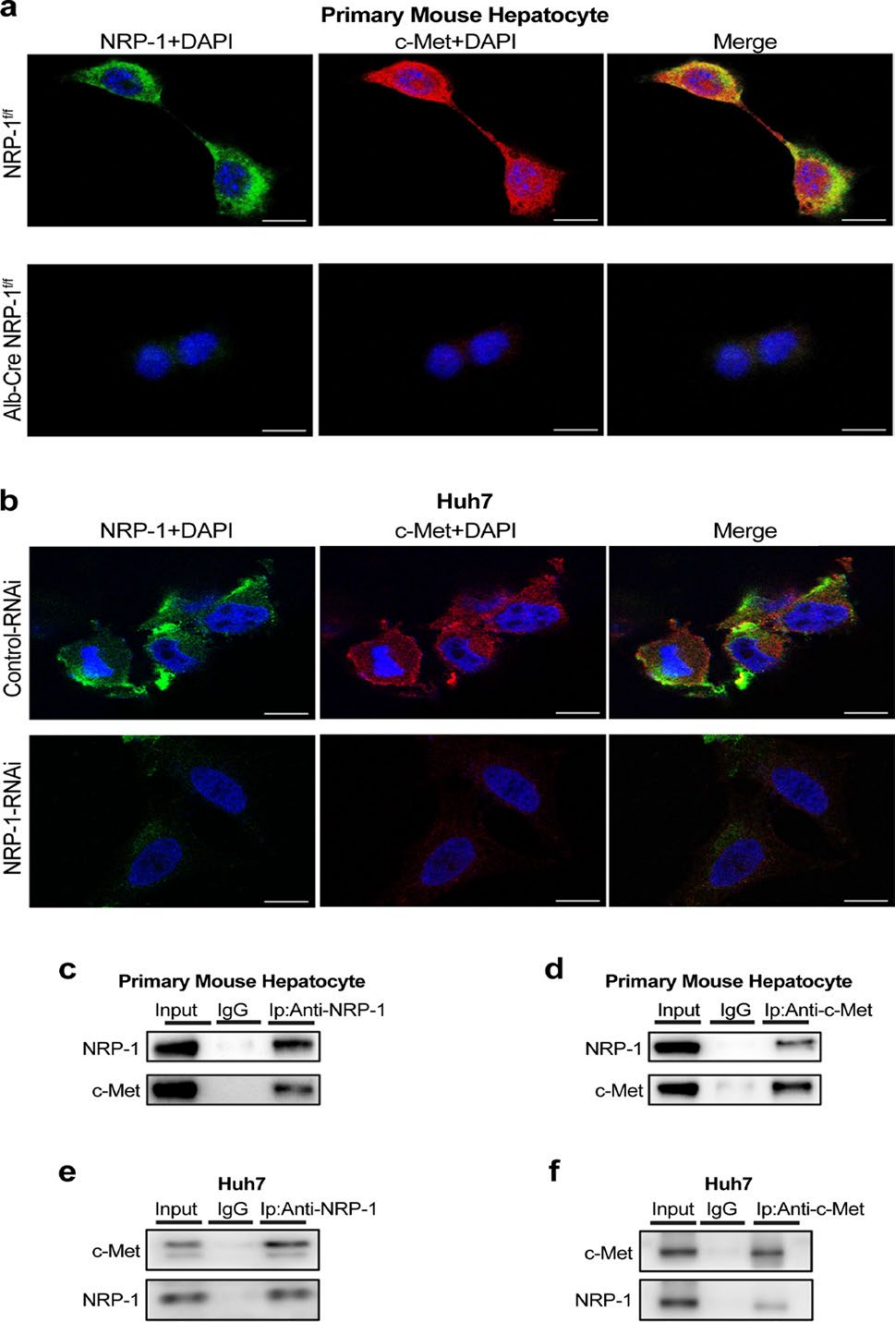
NRP-1 binds to c-Met as a co-receptor. (a) Immunofluorescence images of primary hepatocytes from NRP-1^f/f^ and Alb-Cre NRP-1^f/f^ mice show NRP-1 (green) and c-Met (red) colocalized on the cell surface. Nuclei are stained blue; scale bar: 20 μm. (b) Immunofluorescence images of Huh7 cells transfected with NRP-1-RNAi or vehicle lentivirus show NRP-1 and c-Met localization. (c–d) Co-immunoprecipitation (Co-IP) and immunoblotting confirm the interaction between c-Met and NRP-1 in primary hepatocytes from NRP-1^f/f^ mice. (e–f) Co-IP and immunoblotting confirm the interaction between c-Met and NRP-1 in Huh7 cells

Given the co-expression and co-localization of NRP-1 and c-Met in hepatocytes, we further investigated their interaction. Immunofluorescence analysis confirmed their colocalization on the cell membrane of both primary mouse hepatocytes and Huh7 cells (Fig. 4a–b), suggesting that NRP-1 may promote liver fibrosis as a c-Met co-receptor. Co-immunoprecipitation (Co-IP) assays demonstrated NRP-1 and c-Met binding in hepatocytes to confirm this interaction. Using NRP-1 antibodies, Co-IP assays in primary mouse hepatocytes and Huh7 cells confirmed c-Met’s association with NRP-1 in vitro (Fig. 4c, e). Conversely, c-Met Co-IP assays confirmed its interaction with NRP-1 (Fig. 4d, f).

### NRP-1 promoted liver fibrosis via the Met/ERK pathway in hepatocytes

**Fig. 5:**
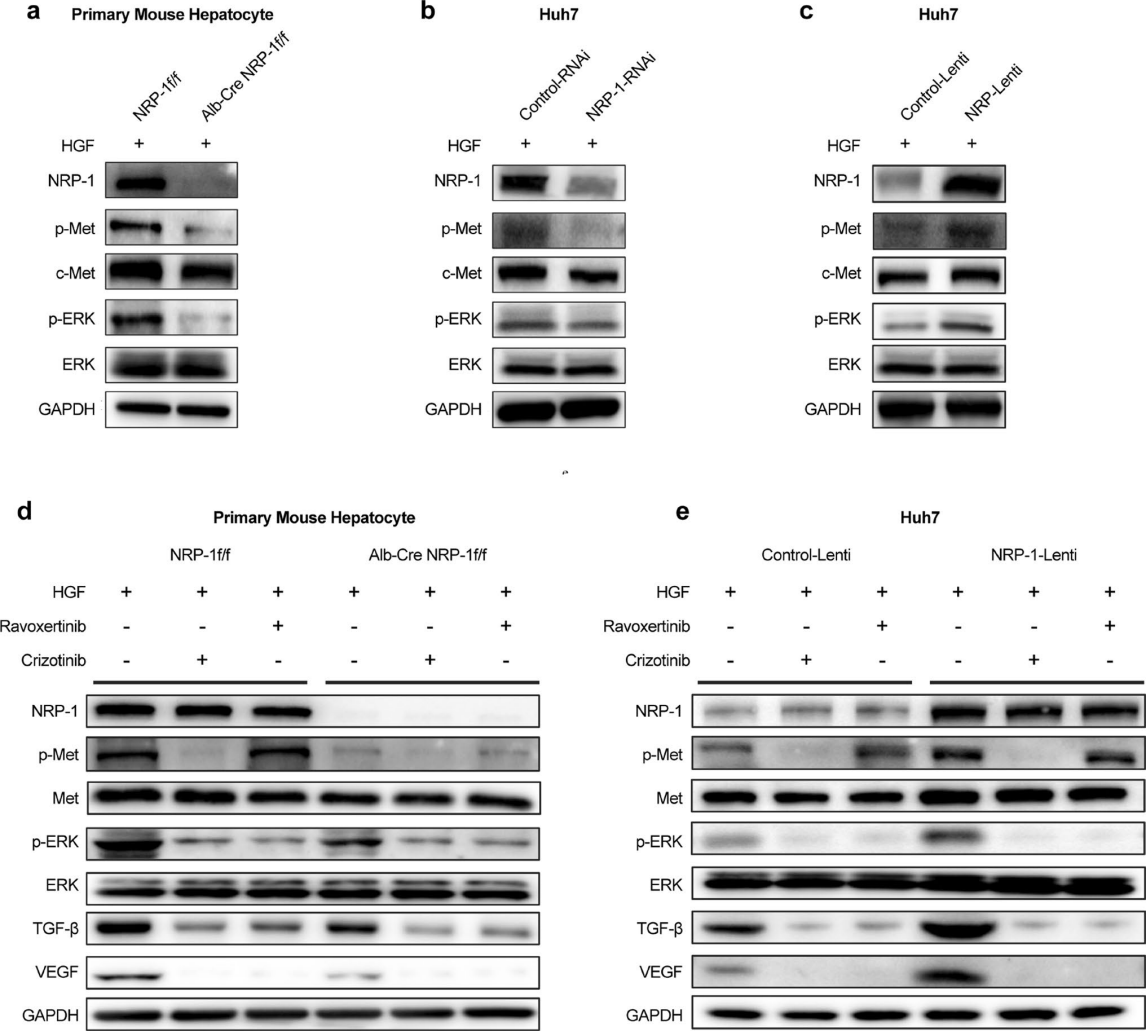
NRP-1 enhances c-Met phosphorylation and downstream ERK pathway activity. (a) The Knockout of NRP-1 significantly reduced ERK phosphorylation and the secretion of TGF-β and VEGF. (b) NRP-1 knockdown similarly decreased ERK phosphorylation and the secretion of TGF-β and VEGF. (c) WB analysis showed that NRP-1 overexpression markedly increased ERK phosphorylation and enhanced TGF-β and VEGF release. (d) HGF, Ravoxertinib (an ERK inhibitor), and Crizotinib (a c-Met inhibitor) were added to primary hepatocytes from NRP-1^f/f^ and Alb-Cre NRP-1^f/f^ mice. Western blotting was used to evaluate the expression of TGF-β, VEGF, and components of the c-Met/ERK signaling pathway. (e) WB analysis was performed on TGF-β, VEGF, and key c-Met/ERK signaling proteins in the NRP-1-Lenti and Control-Lenti groups

Next, we explored the signaling pathways downstream of c-Met regulated by NRP-1 in hepatocytes. NRP-1 deletion significantly reduced ERK phosphorylation in primary mouse hepatocytes (Fig. 5a), and NRP-1 knockdown similarly reduced ERK phosphorylation in Huh7 cells (Fig. 5b). In contrast, NRP-1 overexpression markedly increased ERK phosphorylation in Huh7 cells following HGF treatment in vitro (Fig. 5c).

To confirm this mechanism, we used c-Met and ERK inhibitors. WB showed that crizotinib, a c-Met inhibitor, reduced ERK phosphorylation and suppressed TGF-β and VEGF synthesis (Fig. 5d–e). Additionally, NRP-1 enhanced TGF-β and VEGF release despite ERK pathway inhibition.

### NRP-1 expression was elevated by HGF via transcription factor RARA in hepatocytes

Beyond its role as a co-receptor for HGF/c-Met, NRP-1 expression was upregulated by HGF in primary hepatocytes and Huh7 cells (Fig. 3g, j, m, p, s, v), indicating that HGF activates c-Met and induces NRP-1 expression, amplifying fibrotic signaling.

To explore the mechanism underlying HGF-induced NRP-1 upregulation, we analyzed transcription factors predicted to bind the NRP-1 promoter using the UCSC Genome Browser and the JASPAR database. RARA emerged as a key regulator.

**Fig. 6:**
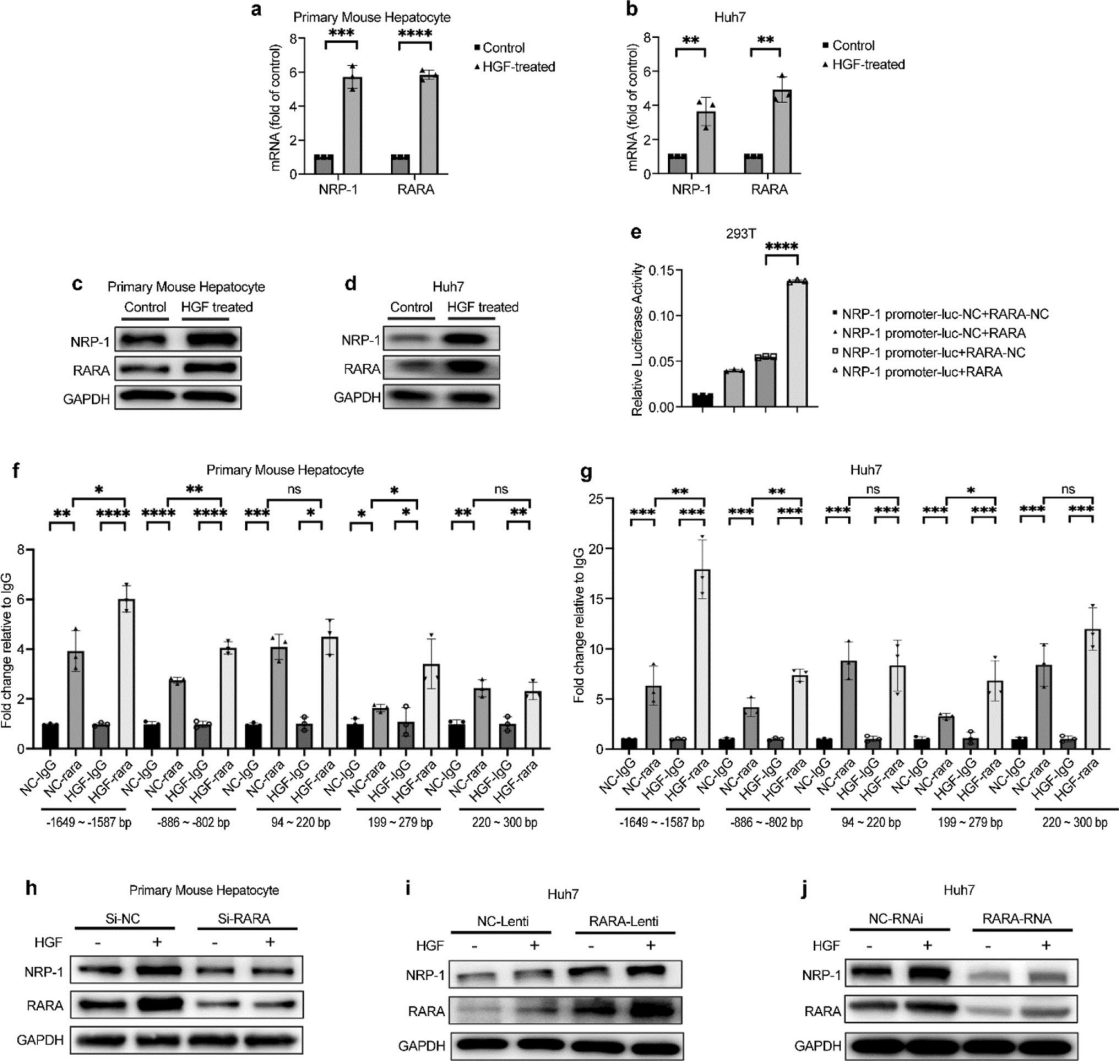
NRP-1 expression is upregulated by HGF via the transcription factor RARA in hepatocytes. (a–d) HGF treatment significantly increased the mRNA and protein levels of RARA and NRP-1 in primary mouse hepatocytes and Huh7 cells. (e) A dual-luciferase reporter assay in 293 T cells validated the interaction between the NRP-1 promoter and RARA. (f–g) ChIP-qPCR analysis revealed enhanced RARA binding to the NRP-1 promoter in primary mouse hepatocytes and Huh7 cells following HGF stimulation, compared to NC-RARA and NC-IgG controls. (h) SiRNA-mediated RARA knockdown (Si-RARA) in primary mouse hepatocytes reduced RARA and NRP-1 expression, which was partially restored by HGF treatment. (i) Lentivirus-mediated RARA knockdown (RARA-RNAi) in Huh7 cells significantly decreased RARA and NRP-1 expression, with HGF stimulation restoring protein levels. (j) WB analysis of RARA and NRP-1 protein expression in the Lenti-NRP-1 and Lenti-Control groups, with or without HGF treatment. *p < 0.05, **p < 0.01, ***p < 0.005, ****p < 0.001

HGF significantly increased RARA and NRP-1 expression in primary hepatocytes and Huh7 cells, confirmed by qRT-PCR (Fig. 6a, b) and WB (Fig. 6c, d). A dual-luciferase reporter assay confirmed the interaction between RARA and the NRP-1 promoter (Fig. 6e). JASPAR-based predictions and designed targeted primers were used to define RARA-binding sites (Online Resource 1). ChIP-PCR confirmed RARA binding to the NRP-1 promoter in both hepatocyte types, with binding enhanced by HGF (Fig. 6f, g).

To further assess RARA’s role in NRP-1 regulation, we transfected primary mouse hepatocytes with Si-RARA targeting RARA (siRNA RARA) or negative control (Si-NC) and employed lentivirus-mediated RARA knockdown (RARA-RNAi) and overexpression (Lenti-RARA) in Huh7 cells. RARA knockdown abolished HGF-induced NRP-1 upregulation; overexpression significantly increased NRP-1 levels (Fig. 6h–j).

## Discussion

**Fig. 7:**
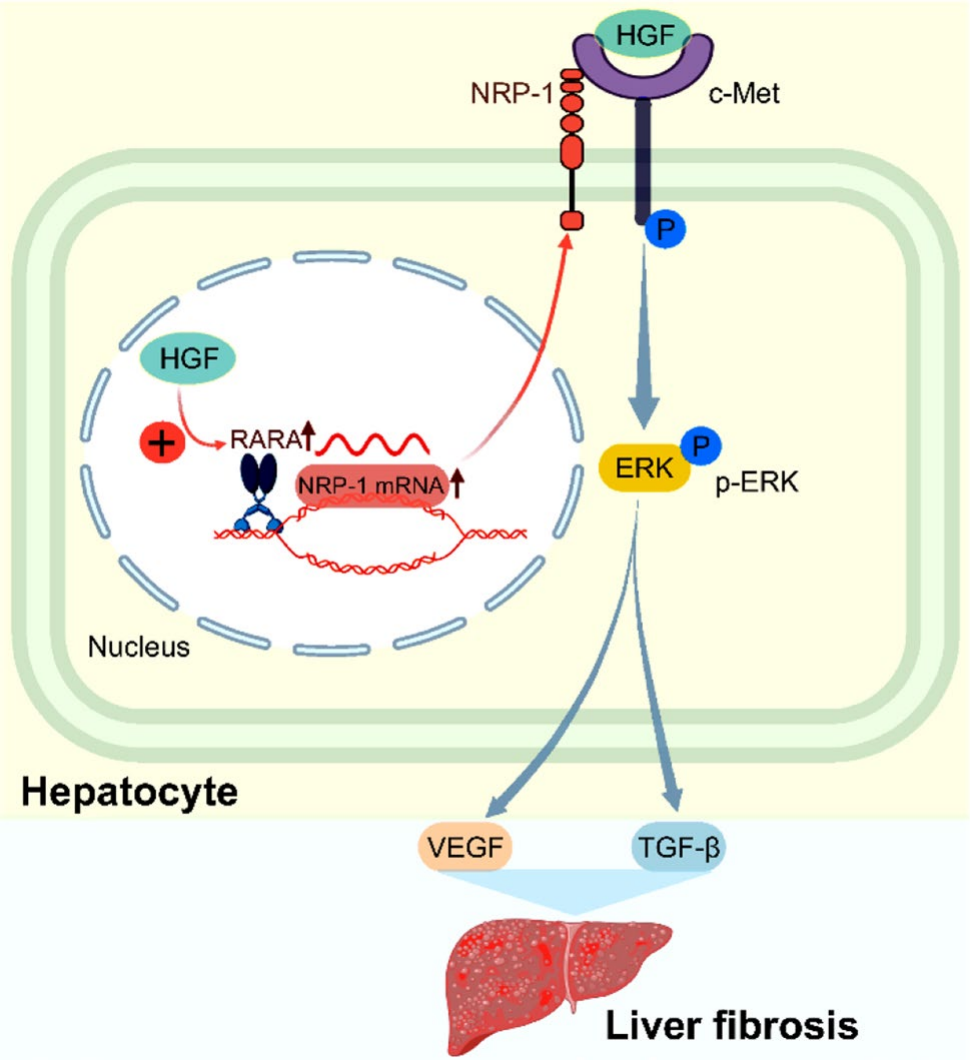
Hepatocyte growth factor upregulates neuropilin-1 expression via retinoic acid receptor alpha, exacerbating liver fibrosis

Liver fibrosis, a precursor to cirrhosis and hepatocellular carcinoma, represents a potentially reversible stage of CLD. In this study, we investigated whether NRP-1 and the HGF/c-Met pathways interact to drive the progression of liver fibrosis. Our findings underscore the critical role of hepatocyte NRP-1 in fibrosis progression. In vivo, NRP-1 inhibition reduced ECM deposition and the expression of pro-fibrotic factors. In vitro, HGF-induced NRP-1 upregulation via RARA activated c-Met/ERK signaling, promoting fibrosis (Fig. 7). These findings strengthen the pathophysiological relevance of the HGF/RARA/NRP-1 axis in liver fibrosis and highlight hepatocyte NRP-1 as a key therapeutic target for fibrosis intervention.

Hepatocyte NRP-1, whose expression is upregulated by HGF by binding the transcription factor RARA to its potential promoter regions, promotes liver fibrosis via the c-Met signaling pathway.

Hepatocytes are the first cells to initiate angiogenesis in liver fibrosis [5, 9]. Impaired hepatocyte function is a key driver of liver fibrosis dynamics [34]. Hepatocytes are also the primary parenchymal cells in the liver, constituting approximately 60% of all liver cells. During liver fibrosis, these cells become a key source of profibrotic and proangiogenic factors, activating HSCs and HSECs, contributing to the progression of fibrosis and abnormal angiogenesis [35]. Our study confirms that NRP-1 is predominantly expressed in hepatocytes and plays a central role in promoting fibrosis through the secretion of TGF-β and VEGF. We isolated primary liver cells from mice and analyzed NRP-1 expression across hepatocytes, HSCs, Kupffer cells, and LSECs. While fibrosis-induced NRP-1 upregulation occurred in all liver cell types, hepatocytes exhibited the highest expression levels. TGF-β stimulation increased NRP-1 expression in HSCs but at significantly lower levels than hepatocytes. These findings suggest that although other liver cells may contribute to fibrosis, hepatocyte-derived NRP-1 is dominant in driving fibrosis progression. These findings confirm that hepatocyte-derived NRP-1 is a dominant profibrotic factor.

Hepatocyte-specific NRP-1 knockout in CCl₄- and NASH-induced fibrosis models reduced fibrosis severity, collagen deposition, and angiogenesis, along with lower TGF-β and VEGF secretion. This confirms that NRP-1 is a critical profibrotic factor, regardless of the fibrosis etiology. Additionally, NRP-1 deletion protected hepatocytes from injury, as evidenced by reduced serum ALT and AST levels following repeated CCl₄ injections. These findings suggest that NRP-1 promotes fibrosis via extracellular matrix remodeling, pathological angiogenesis, and hepatocyte damage through TGF-β and VEGF secretion.

Given that hepatocytes stimulate the activation of HSCs and HSECs, driving liver fibrosis and cirrhosis progression, elucidating the function of NRP-1 in hepatocytes could potentially impede or reverse fibrosis in its early stages. This approach targets multiple pathogenic mechanisms simultaneously, positioning NRP-1 as a promising therapeutic target for liver fibrosis.

The HGF/c-Met axis is crucial in CLD pathogenesis [24, 30]. HGF is secreted by mesenchymal cells and significantly increases after liver injury [36]. Our investigation revealed that elevated HGF secretion was associated with liver fibrosis. HGF binds to its receptor, c-Met, and transmits signals to cells, activating the intrinsic kinase function of c-Met. Since the HGF/c-Met pathway and NRP-1 are both crucial in CLD, we investigated how NRP-1 modulates the HGF/c-Met axis. We demonstrated a notable upregulation of NRP-1 expression in hepatocytes, which correlated positively with to c-Met in the context of liver fibrosis. Previous research has shown that NRP-1 is a well-established co-receptor on cell surfaces that interacts with other receptor tyrosine kinases and plays a crucial role in the progression of various diseases, including its interaction with c-Met in colorectal cancer, the epidermal growth factor receptor in gastric cancer, and VEGFR2 and PDGFR in liver fibrosis [17, 18, 37, 38]. Our subsequent investigations revealed that hepatocyte NRP-1 functions as a co-receptor for c-Met during liver fibrosis. As a transmembrane protein, NRP-1 is characterized by a relatively short intracellular region lacking enzymatic functions. Therefore, it is widely acknowledged that NRP-1 facilitates important physiological reactions by forming a complex with c-Met that depends on HGF [13]. Furthermore, our novel experimental results demonstrated that NRP-1 not only facilitates HGF/c-Met signaling but is also transcriptionally and translationally upregulated by HGF in hepatocytes, reinforcing its role in fibrosis progression.

The canonical mechanism of c-Met activation is illustrated by the binding of HGF to the c-Met receptor, which leads to the homodimerization of c-Met and the subsequent autophosphorylation of its cytoplasmic domain, activating downstream signaling cascades. Beyond the canonical activation of c-Met by HGF binding, NRP-1 act as a co-receptor, further stimulating c-Met, exacerbating liver fibrosis. Additionally, previous studies have shown that c-Met can be activated through non-canonical pathways involving integrin α6β4, the human epidermal growth factor receptor, and focal adhesion kinase [39–41]. Our findings extend this by showing that HGF increases NRP-1 expression, enhancing TGF-β and VEGF secretion, linking mesenchymal-derived HGF, NRP-1 upregulation, and c-Met signaling in fibrosis.

We investigated the molecular mechanisms by which HGF regulates NRP-1 expression. The NRP-1 promoter sequence was identified using the National Center for Biotechnology Information database. We analyzed potential transcription factors that could interact with the NRP-1 promoter, identifying those with a minimum score of 600 on the UCSC Genome website. The binding sites of these transcription factors were predicted using the most recent operational handbook on the JASPAR website [42–45]. Our results demonstrate that HGF increases NRP-1 expression by activating the transcription factor RARA, which is a component of the promyelocytic leukemia/RARA oncoprotein. This protein disrupts transcriptional programs, hindering hematopoietic progenitor cell regeneration and leading to leukemia development [46, 47]. RARA binding to the NRP-1 promoter was verified by a dual-luciferase assay, which identified three potential binding sites: −886 to −802, −1649 to −1587, and 199 to 279 base pairs. Enhanced RARA binding at these sites was observed following HGF stimulation, potentially increasing NRP-1 transcription. In cell models of RARA overexpression and knockdown, overexpression increased NRP-1 expression, while knockdown reduced it. C-Met activates several intracellular signaling pathways, including p38, ERK1/2, and c-Jun N-terminal kinase, which regulate cell proliferation, invasion, and metastasis [30, 48].

This study demonstrated that c-Met phosphorylation triggers the ERK signaling cascade. However, inhibiting p38 and c-Jun N-terminal kinase did not significantly affect TGF-β and VEGF production, suggesting that these pathways are not involved in their regulation. Previous research has linked ERK activation to cell proliferation, invasion, and metastasis [49]. Our findings show that the c-Met/ERK pathway also increases TGF-β and VEGF secretion.

In conclusion, NRP-1 is a pivotal mediator in the pathogenesis of liver fibrosis, with its expression and function intricately linked to disease progression. Targeting the HGF/RARA/NRP-1 axis may offer a promising therapeutic strategy for managing liver fibrosis, potentially by developing drugs that modulate NRP-1 expression or function. These interventions could be particularly effective in the early stages of liver fibrosis, underscoring the importance of NRP-1 as a therapeutic target for future clinical applications.

## Supplementary Information

Below is the link to the electronic supplementary material.

Supplementary file1 (DOCX 1798 KB)

## Data Availability

The datasets used and/or analyzed during the current study are available from the corresponding author upon reasonable request.
