# Supplementary material for: Interaction of neuropilin-1 and hepatocyte growth factor/C-Met pathway in liver fibrosis progression in hepatocyte-specific NRP-1 knockout mice
Source: J Gastroenterol. 2025 May 26;60(8):1000–13. doi: 10.1007/s00535-025-02262-8 (PMC12289806; doi:10.1007/s00535-025-02262-8)
Supplement: Supplementary file 1 — Supplementary file1 (DOCX 1798 KB) [file 535_2025_2262_MOESM1_ESM.docx]

**Supplementary Materials**

*Journal of Gastroenterology*

**Interaction of Neuropilin-1 and Hepatocyte Growth Factor/c-Met Pathway in Liver Fibrosis Progression in Hepatocyte-Specific NRP-1 Knockout Mice**

Han Ding,^1^ Huanran Lv,^1^ Minghao Sui,^2^ Xinyu Wang,^1^ Yanning Sun,^3^ Miaomiao Tian,^1^ Shujun Ma,^1^ Yuchan Xue,^1^ Miao Zhang,^4^ Xin Wang,^5^ Jianni Qi,^6^ Le Wang,^7^ Qiang Zhu^8^

^1^Department of Gastroenterology, Shandong Provincial Hospital Affiliated to Shandong First Medical University, No. 324, Jingwu Weiqi Road, Huaiyin District, Jinan City, Shandong Province, China

^2^Department of Gastroenterology, Shandong Provincial Hospital, Cheeloo College of Medicine, Shandong University, No. 324, Jingwu Weiqi Road, Huaiyin District, Jinan City, Shandong Province, China

^3^Urology Department, Shandong Provincial Hospital, Cheeloo College of Medicine, Shandong University, No. 324, Jingwu Weiqi Road, Huaiyin District, Jinan City, Shandong Province, China

^4^Department of Radiology, Shandong Provincial Hospital Affiliated to Shandong First Medical University, No. 324, Jingwu Weiqi Road, Huaiyin District, Jinan City, Shandong Province, China

^5^Department of Ultrasound, Shandong Provincial Hospital Affiliated to Shandong First Medical University, No. 324, Jingwu Weiqi Road, Huaiyin District, Jinan City, Shandong Province, China

^6^Department of Key Laboratory, Shandong Provincial Hospital Affiliated to Shandong First Medical University, No. 324, Jingwu Weiqi Road, Huaiyin District, Jinan City, Shandong Province, China

^7^Department of Geriatrics, Department of Geriatric Gastroenterology, Shandong Provincial Hospital Affiliated to Shandong First Medical University, No. 324, Jingwu Weiqi Road, Huaiyin District, Jinan City, Shandong Province, China

^8^Department of Infectious Diseases, Shandong Provincial Hospital Affiliated to Shandong First Medical University, No. 324, Jingwu Weiqi Road, Huaiyin District, Jinan City, Shandong Province, China

**Online Resource 1**. List of primers and siRNA sequences used in the experiments

| Primer (Human) | Sequence (5’-3’) |
| --- | --- |
| GAPDH F | GCACCGTCAAGGCTGAGAAC |
| GAPDH R | TGGTGAAGACGCCAGTGGA |
| NRP-1F | TCAACCCTCACTTCGATTTGGA |
| NRP-1R | GCACCATGTGTTTCGTAGTCAGAGA |
| HGF F | GCTATCGGGGTAAAGACCTACA |
| HGF R | CGTAGCGTACCTCTGGATTGC |
| c-Met F | AGCAATGGGGAGTGTAAAGAGG |
| c-Met R | CCCAGTCTTGTACTCAGCAAC |
| α-SMA F | ATTGCCGACCGAATGCAGA |
| α-SMA R | ATGGAGCCACCGATCCAGAC |
| TGF-β F | GGCCAGATCCTGTCCAAGC |
| TGF-β R | GGCCAGATCCTGTCCAAGC |
| VEGF F | AGGGCAGAATCATCACGAAGT |
| VEGF R | AGGGTCTCGATTGGATGGCA |
| RARA F | AAGCCCGAGTGCTCTGAGA |
| RARA R | TTCGTAGTGTATTTGCCCAGC |
| Human NRP-1（-1649~-1587）F | TCGAGCAGTTACCATCCAGT |
| Human NRP-1（-1649~-1587）R | TCCCTGAGTCGGATTGGAAC |
| Human NRP-1（-886~-802）F | GGCTGGAAAATGCACACTCA |
| Human NRP-1（-886~-802）R | GAGGGAGACTTGAGCTGTGT |
| Human NRP-1（94~220）F | GTGTGTGTAGACGCCAATCC |
| Human NRP-1（94~220）R | AAGAAACAGGTTGCGGTCAC |
| Human NRP-1（199~279）F | GTGACCGCAACCTGTTTCTT |
| Human NRP-1（199~279）R | GTTTTCCGAGAAGTGCTGCA |
| Human NRP-1（220~300）F | GTGACCGCAACCTGTTTCTT |
| Human NRP-1（220~300）R | GTTTTCCGAGAAGTGCTGCA |

| Primer (Mouse) | Sequence (5’-3’) |
| --- | --- |
| GAPDH F | AAATGGTGAAGGTCGGTGTGAAC |
| GAPDH R | CAACAATCTCCACTTTGCCACTG |
| NRP-1F | AGCTACTGGGCTGTGAAGTGGAA |
| NRP-1R | TGTGAGCTGGAAGTCATCACCTG |
| α-SMA F | CCAGCTATGTGTGAAGAGGAAGA |
| α-SMA R | TTGGTGATGATGCCGTGTTCTAT |
| Alb-cre F | TAGGAACCAATGAAATGCGAGGT |
| Alb-cre R | AACCAGCGTTTTCGTTCTGC |
| NRP-1 flox F | AAGGAGTGGCACAGCATCTT |
| NRP-1 flox R | TCACACCCAAACTTCCTTCC |
| HGF F | GCAGTACCCTCACAAGCATGATA |
| HGF R | GACACGTCACACTTGGGAATTTG |
| c-Met F | ACCCTGAAGCAGTTAAAGGTGAA |
| c-Met R | GAGACTGCTTGCTTCCACTCTAT |
| VEGF F | AAACGAACGTACTTGCAGATGTG |
| VEGF R | TCTTCCTTCATGTCAGGCTTTCT |
| TGF-β F | GCGGACTACTATGCTAAAGAGGT |
| TGF-β R | GCTTCCCGAATGTCTGACGTATT |

| SiRNA (Mouse) | Sequence (5’-3’) |
| --- | --- |
| RARA | CCGCTTTGGAATGGCTCAAACCACT |

_GAPDH: Glyceraldehyde-3-Phosphate Dehydrogenase, NRP-1: Neuropilin-1, HGF: Hepatocyte Growth Factor, c-Met: C-Met Proto-Oncogene (also known as Hepatocyte Growth Factor Receptor), α-SMA: Alpha-Smooth Muscle Actin, TGF-β: Transforming Growth Factor Beta, VEGF: Vascular Endothelial Growth Factor, RARA: Retinoic Acid Receptor Alpha, Alb-cre: Albumin-Cre Recombinase, NRP-1 flox: Floxed Neuropilin-1 (for conditional knockout studies)_

**Online Resource 2**. Antibodies and small molecule inhibitors used in the experiment

Antibodies:
NRP-1 (ab81321), TGF-β1 (ab215715), VEGF-A (ab46154), α-SMA (ab7817), and CD31 (ab28364) were purchased from Abcam (Cambridge, UK).
C-Met (8198T), phosphorylated C-Met (p-Met, 3077T), extracellular signal-regulated kinase (ERK, 4695T), phosphorylated ERK (4370T), P38 (9212S), phospho-P38 (4511T), and RARA (62294S) were sourced from Cell Signaling Technology (Danvers, MA, USA).

Small Molecule Inhibitors:
HGF and TGF-β were purchased from PeproTech (Cranbury, NJ, USA).
c-Met inhibitor (Crizotinib, S1068) and ERK inhibitor (Ravoxertinib, S7554) were obtained from Selleck Chemicals (Houston, TX, USA).

Secondary Antibodies for Immunofluorescence:
Alexa Fluor® 488 and 594-conjugated secondary antibodies were provided by Proteintech (Wuhan, China).

**Online Resource 3.** Supplementary Fig. 1

**
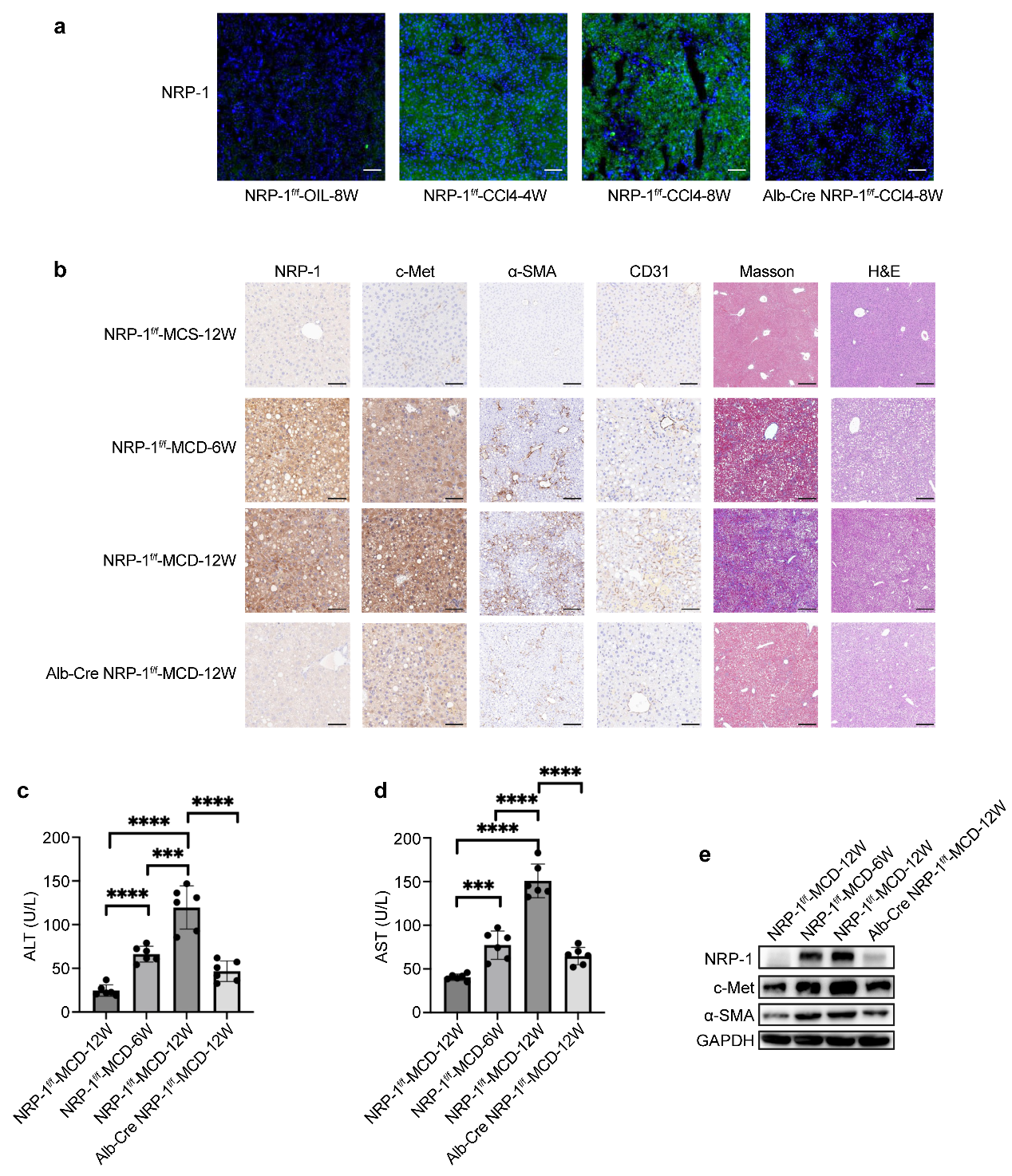
**

**Supplementary Fig. 1 NRP-1 knockout ameliorates liver fibrosis in hepatocyte-specific NRP-1-knockout mice induced by an MCD diet**

(a) Immunofluorescence analysis of NRP-1 expression, with DAPI (blue) marking nuclei and NRP-1 (green) marking hepatocytes. Scale bar: 200 μm. (b) Representative histological and immunohistochemical staining of liver tissues from MCD diet-fed mice, showing NRP-1 (×400, scale bar: 50 μm), c-Met (×400, scale bar: 50 μm), α-SMA (×200, scale bar: 100 μm), and CD31 (×400, scale bar: 50 μm). Hematoxylin and eosin (H&E) and Masson’s trichrome staining (×100, scale bar: 200 μm) assessed fibrosis severity and collagen accumulation. (c-d) Serum ALT and AST levels increased with fibrosis severity in MCD-fed mice but significantly decreased following hepatocyte-specific NRP-1 knockout. (e) Western blot analysis of NRP-1, c-Met, and α-SMA expression in normal, MCD-induced fibrosis, and NRP-1 knockout MCD-fibrosis groups.
